# E-Beam Effects on Poly(Xylitol Dicarboxylate-co-diol Dicarboxylate) Elastomers Tailored by Adjusting Monomer Chain Length

**DOI:** 10.3390/ma14071765

**Published:** 2021-04-02

**Authors:** Marta Piątek-Hnat, Kuba Bomba, Janusz P. Kowalski-Stankiewicz, Jakub Pęksiński, Agnieszka Kozłowska, Jacek G. Sośnicki, Tomasz J. Idzik, Beata Schmidt, Krzysztof Kowalczyk, Marta Walo, Grzegorz Mikołajczak, Agnieszka Kochmańska

**Affiliations:** 1 Faculty of Chemical Technology and Engineering, West Pomeranian University of Technology, Piastów Ave. 42, 71-065 Szczecin, Poland; kubabomba4@gmail.com (K.B.); agak@zut.edu.pl (A.K.); 2 Department of Computer Sciences in Medicine & Education Quality Evaluation, Pomeranian Medical University in Szczecin, Żołnierska St. 54, 71-210 Szczecin, Poland; janus@pum.edu.pl; 3 Faculty of Electrical Engineering, West Pomeranian University of Technology, Sikorskiego Ave. 37, 71-313 Szczecin, Poland; jakub.peksinski@zut.edu.pl (J.P.); Grzegorz.Mikolajczak@zut.edu.pl (G.M.); 4 Department of Organic and Physical Chemistry, Faculty of Chemical Technology and Engineering, West Pomeranian University of Technology, Piastów Ave. 42, 71-065 Szczecin, Poland; jacek.sosnicki@zut.edu.pl (J.G.S.); tomasz.idzik@zut.edu.pl (T.J.I.); 5 Department of Chemical Organic Technology and Polymeric Materials, Faculty of Chemical Technology and Engineering, West Pomeranian University of Technology, Piastów Ave. 42, 71-065 Szczecin, Poland; Beata.Schmidt@zut.edu.pl (B.S.); Krzysztof.Kowalczyk@zut.edu.pl (K.K.); 6 Laboratory for Measurements of Technological Doses, Institute of Nuclear Chemistry and Technology, Dorodna St. 16, 03-195 Warsaw, Poland; M.Walo@ichtj.waw.pl; 7 Department of Materials Technology, West Pomeranian University of Technology, 70-310 Szczecin, Poland; akochmanska@zut.edu.pl

**Keywords:** xylitol, biodegradable elastomers, radiation modification, e-beam, mechanical and thermal properties

## Abstract

Poly(xylitol dicarboxylate-co-diol dicarboxylate) elastomers can by synthesized using wide variety of monomers with different chain lengths. Obtained materials are all biodegradable, thermally stable elastomers, but their specific properties like glass transition temperature, degradation susceptibility, and mechanical moduli can be tailored for a specific application. Therefore, we synthesized eight elastomers using a combination of two dicarboxylic acids, namely suberic and sebacic acid, and four different diols, namely ethanediol, 1,3-propanediol, 1,4-buanediol, and 1,5-pentanediol. Materials were further modified by e-beam treatment with a dose of 100 kGy. Materials both before and after radiation modification were tested using tensile tests, gel fraction determination, ^1^H NMR, and ^13^C NMR. Thermal properties were tested by Differential Scanning Calorimetry (DSC), Dynamic Thermomechanical Analysis (DMTA) and Thermogravimetric Analysis (TGA). Degradation susceptibility to both enzymatic and hydrolytic degradation was also determined.

## 1. Introduction

Due to the high consumption of commodity polymers and the difficulty of their recycling, it is of great importance to develop materials that are not only biodegradable but also at least partially based on monomers obtainable from renewable sources. There is an additional advantage if such polymers react well to radiation modification, which allows to improve their properties in a time-, energy-, and space-saving way that is easy to control and operate [1]. Such materials described in the literature are polylactide [2,3,4], polycaprolacone, [5,6], poly(butylene succinate) [7], poly(hydroxyalkanoate) [8], poly-(R)-3-hydroxybutyrate [9], and sugar-alcohol-based polyesters [10,11,12]. Materials belonging to this last group are elastomers synthesized by the polycondensation reaction of a sugar alcohol, such as xylitol, and a dicarboxylic acid, which leads to obtaining poly(polyol dicarboxylate) polyesters [13,14,15]. The temperature of glass transition, stress and elongation at break, and time of degradation of those materials can be fine-tuned for a specific use, while their most important characteristics, namely biodegradability and elastomeric behavior, are preserved. This can be done by altering the length of dicarboxylic acid used as monomer [15,16], the hydroxyl group content of sugar alcohol [13,14], stoichiometric ratio of monomers [13,17], or reaction temperature [18]. Most extensively described poly(polyol dicarboxylate) polyesters are materials based on sebacic acid and xylitol [19,20,21], or glycerol [18,22,23,24,25].

Glycerol-based elastomers in particular were extensively tested in terms of possible biomedical applications. Other than the possible application of poly(glycerol sebacate) (PGS) as drug carrier [26], a wide range of potential uses in tissue engineering were discussed in the literature. PGS can be used as scaffold for bone tissue regeneration [23,27], myocardial tissue regeneration [18], hollow conduit guides for treatment of neural defects [22], delivering retinal progenitor cell to the subretinal area [28], treatment of defects in cartilage tissue [25], and regeneration of blood vessels [24].

Poly(xylitol sebacate) (PXS) elastomers also hold significant promise in the biomedical field, for example, tissue-like materials obtained by core/shell electrospinning PXS with poly(vinyl alcohol) as a sacrificial component [20,21].

The properties of sugar-alcohol-based elastomers can be further improved by utilizing a diol as a third monomer, which leads to a poly(polyol dicarboxylate-co-diol dicarboxylate) product. The properties of materials belonging to this group can also by tailored by utilizing different polycondensation times [29], by changing the chain length of dicarboxylic acid [30] or diol [12,31] used for the synthesis, or by changing the hydroxyl group content of the polyol [10,32,33]. Further fine-tuning of their properties can be conducted with 50 to 150 kGy e-beam treatment with doses ranging from 50 to 150 kGy, each leading to slightly different end-product [10,32].

Poly(polyol dicarboxylate-co-diol dicarboxylate) elastomers exhibit a wide range of obtainable characteristics, and modifying those materials with radiation leads to beneficial results. Therefore, we decided to synthesize and perform an e-beam treatment on 8 materials based on xylitol, sebacic and suberic acid, and four different diols as monomers. A radiation dose of 100 kGy was chosen based on previous research [10,32]. Most of these materials, to the best of our knowledge, were never synthesized and radiation-modified before. In comparison with our previous work [10,29,30,32,34], an improved synthesis method was used, which allowed to greatly decrease the cross-linking time from 12 days to about 48 h.

Overall, due to the fact that materials presented in this work react well to radiation, they could see a potential application as polymer boluses for radiotherapy [35,36].

## 2. Materials and Methods

### 2.1. Synthesis of Elastomers

Sigma-Aldrich (St. Louis, MO, USA) was the chemical supplier. All ingredients were reagent grade. The 8 poly(xylitol dicarboxylate-diol dicarboxylate) elastomers were synthesized.

Poly(xylitol suberate-co ethylene suberate) (PXESb) was synthesized using xylitol, suberic acid, and ethanediol. Poly(xylitol suberate-co-propylene suberate) (PXPSb) was synthesized using xylitol, suberic acid, and 1,3-propanediol. Poly(xylitol suberate-co-butylene suberate) (PXBSu) was synthesized using xylitol, suberic acid, and 1,4-butanediol. Poly(xylitol suberate-co-pentylene suberate) (PXPeSu) was synthesized using xylitol, suberic acid, and 1,5-pentanediol.

Poly(xylitol sebacate-co-ethylene sebacate) (PXES) was synthesized using xylitol, sebacic acid, and ethanodiol. Poly(xylitol sebacate-co-propylene sebacate) (PXPS) was synthesized using xylitol, sebacic acid, and 1,3-propanediol. Poly(xylitol sebacate-co-butylene sebacate) (PXBS) was synthesized using xylitol, sebacic acid, and 1,4-butanediol. Poly(xylitol sebacate-co-pentylene sebacate) (PXPeS) was synthesized using xylitol, sebacic acid, and 1,5-pentanediol.

The synthesis process consists of three steps: 9 h esterification reaction at 150 °C in nitrogen atmosphere in a vacuum evaporator, 3 h polycondensation reaction at 150 °C in low-pressure-atmosphere (100 mBar) in a vacuum evaporator, and cross-linking reaction of materials cast into silicone forms at 150 °C in low-pressure-atmosphere (100 mBar) in a vacuum dryer. The molar ratio of dicarboxylic acid:xylitol:diol was 2:1:1. No catalyst was used. Synthesis was performed in melt—no solvent was used.

### 2.2. Irradiation

E-beam treatment of cross-linked elastomers was done in the Institute of Nuclear Chemistry and Technology (Warsaw, Poland) with an Elektronika 10/10 linear electron accelerator (NPO, Torij, Russia). Parameters were 10 MeV beam, 360 mA average set current, 0.368 m/min sample movement speed, and 100 kGy split into doses of 25 kGy.

## 3. Experimental Methods

### 3.1. Nuclear Magnetic Resonance Spectroscopy (NMR)

The prepolymer chemical structure was ascertained by ^1^H NMR (at 400.1 MHz) and ^13^C NMR (at 106.6 MHz), with Bruker DPX III HD (Bruker, Rheinstetten, Germany), with 50 mg sample mass, 0.7 mL of CDCl_3_ solvent (deuterated chloroform), and internal reference (Tetramethylsilane-TMS). The results analysis was performed with MestReNova 12.0.3 (Mestrelab, Santiago de Compostela, Spain).

### 3.2. Fourier Transform Infrared Spectroscopy (FTIR)

The elastomer chemical structure (prepolymers, cross-linked materials, and e-beam-treated materials) was ascertained with Alpha Spectrometer Bruker (Bruker, Germany), with 2 cm^−1^ resolution and 4000 to 400 cm^−1^ range. The results analysis was performed with Omnic 7.3 (Thermo Electron Corporation, Waltham, MA, USA).

### 3.3. Differential Scanning Calorimetry (DSC)

The thermal behavior of the elastomers (prepolymers, cross-linked materials, and e-beam-treated materials) was tested with Q2500 DSC instrument (TA instruments, New Castle, DE, USA), with heating range −100 to 100 °C, heating rate 10 °C/min, and in nitrogen atmosphere. The results analysis was performed with TA Instruments Universal Analysis 2000, 3.9a (New Castle, DE, USA).

### 3.4. Dynamic Thermomechanical Analysis (DMTA)

The thermomechanical behavior of the elastomers (cross-linked materials and e-beam-treated materials) was tested with DMA Q800 (TA Instruments, New Castle, DE, USA) with 2 °C/min rate of heating, 1 Hz frequency, and −100 to 100 °C temperature range. The results analysis was performed with TA Instruments Universal Analysis 2000, 3.9a (New Castle, DE, USA).

### 3.5. Mechanical Properties

The mechanical behavior of elastomers before and after e-beam treatment was tested with Instron 36 (Norwood, MA, USA), with a crosshead speed of 100/mm/min, 500 N load cell, in relative humidity of 50%, and at 25 °C, according to the standard PN-EN-ISO 526/1:1996.

### 3.6. Gel Fraction

Gel fraction determination was performed on polymer samples (cross-linked, 0.5 g mass) immersed for 5 days in solvent (tetrahydrofuran, THF). Samples after drying (desiccator, 14 days, 25 °C, lowered pressure) were weighed. The mass-loss calculation was performed with Equation (1):

(1) $$X\;=\;\frac{m_{1}}{m_{0}}\;\times \;100\%$$

where m_1_—sample mass after extraction, m_0_—sample mass before extraction.

### 3.7. Hydrolytic Degradation

Hydrolytic degradation was performed on UV-sterilized (20 min duration, laminar chamber) 10 mm elastomer discs in a 48-well plate, each covered with PBS (phosphate-buffered saline) (Sigma Aldrich, Poznan, Poland) solution (1.5 mL, 7.1 to 7.2 range of pH). The process took 21 days in 37 °C. The solution was changed, and samples were sterilized again every 2 days. Samples after drying (desiccator, 14 days, 25 °C, lowered pressure) were weighed. The mass-loss calculation was performed with Equation (2):

(2) $$D\;=\;\frac{\left(m_{0}\;-\;m_{1}\right)}{m_{0}}\;\times \;100\%$$

where m_0_ is the sample mass pre-degradation, m_1_ is the sample mass post-degradation, and D is the mass loss.

### 3.8. Enzymatic Degradation

Enzymatic degradation was performed on UV-sterilized (20 min duration, laminar chamber) 10 mm elastomer discs in a 48-well plate, each covered with a solution of porcine lipase in PBS (phosphate-buffered saline) (Sigma Aldrich, Poznan, Poland) (1.5 mL, 7.1 to 7.2 range of PH). The process took 21 days, and took place at 37 °C temperature. The solution was changed, and samples were sterilized again every 2 days. Samples after drying (desiccator, 14 days, 25 °C, lowered pressure) were weighed. The mass-loss calculation was performed with Equation (3):

(3) $$D\;=\;\frac{\left(m_{0}\;-\;m_{1}\right)}{m_{0}}\;\times \;100\%$$

where m_0_ is the sample mass pre-degradation, m_1_ is the sample mass post-degradation, and D is the mass loss.

### 3.9. Thermogravimetric Analysis (TGA)

The thermal stability of the non-radiation-modified cross-linked elastomers was tested with Q500 TGA instrument (TA instruments, New Castle, DE, USA), with heating rate of 10 °C/min, heating range of 25 °C to 600 °C and in dry air atmosphere. Instrument was equipped with platinum crucible, and samples weighed about 15 mg each. The results analysis was performed with TA Instruments Universal Analysis 2000, 3.9a (New Castle, DE, USA).

## 4. Results and Discussion

**Figure 1:**
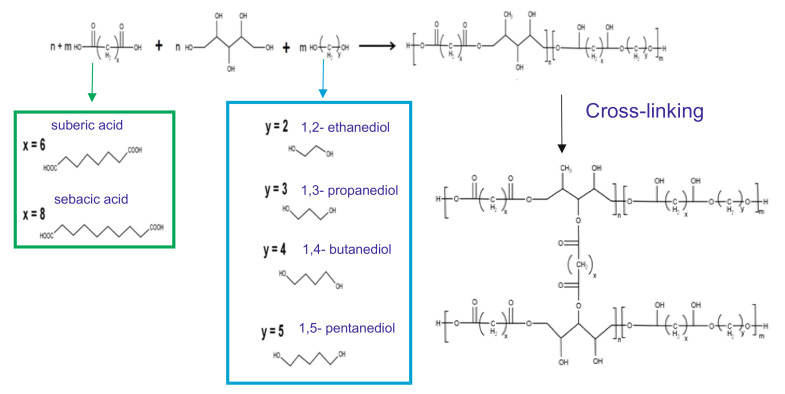
Scheme of the poly(xylitol dicarboxylate-co-diol dicarboxylate) synthesis.

Table 1 summarizes the elastomer properties and composition, and Figure 1 illustrates the polymer structure.

### 4.1. Nuclear Magnetic Resonance Spectroscopy (NMR)

**Figure 2:**
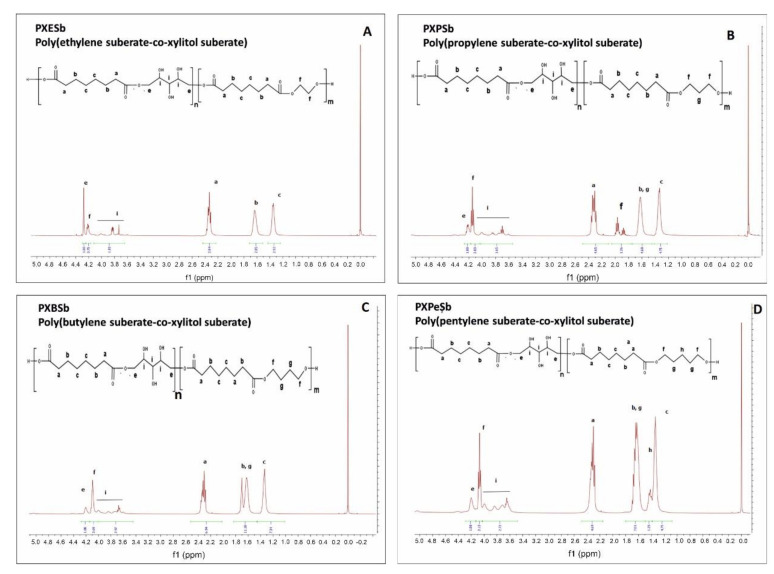
^1^H NMR of following prepolymers: PXESb (**A**), PXPSb (**B**), PXBSb (**C**), PXPeSb (**D**).

**Figure 3:**
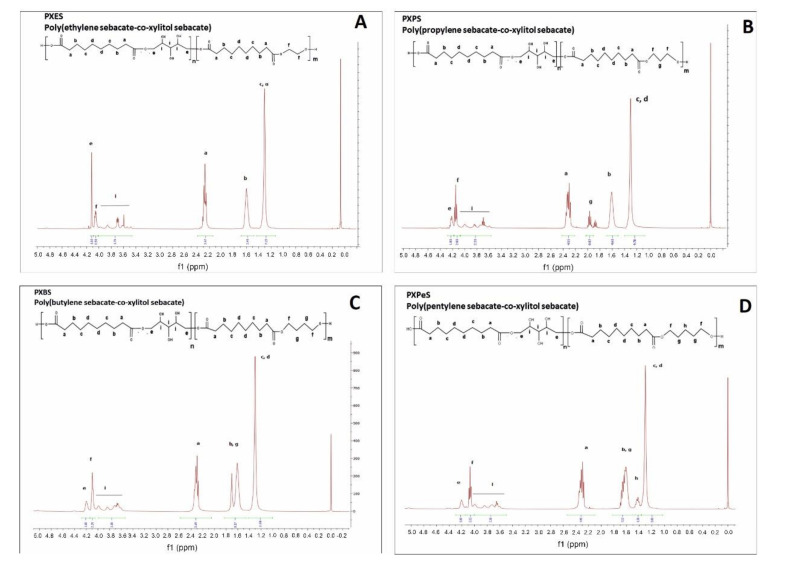
1H NMR of following prepolymers: PXES (**A**), PXPS (**B**), PXBS (**C**), PXPeS (**D**).

**Figure 4:**
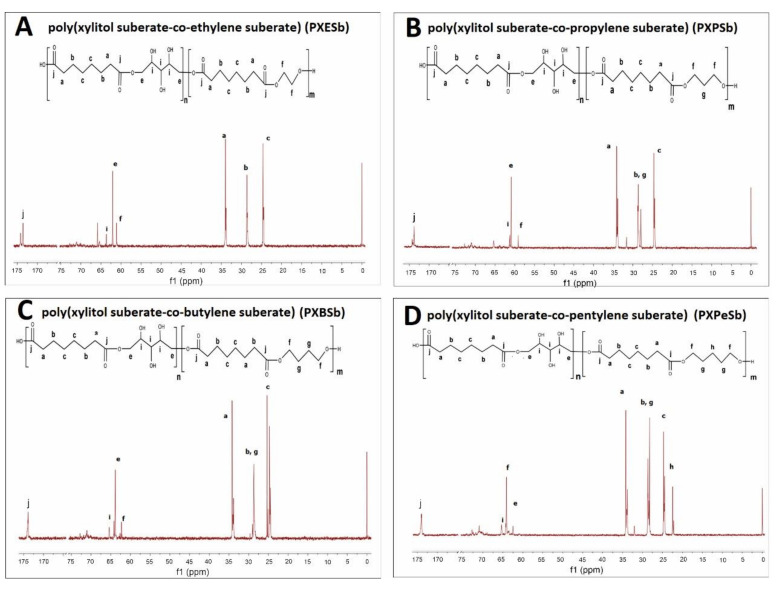
^13^C NMR of following prepolymers: PXESb (**A**), PXPSb (**B**), PXBSb (**C**) PXPeSb (**D**) prepolymers.

**Figure 5:**
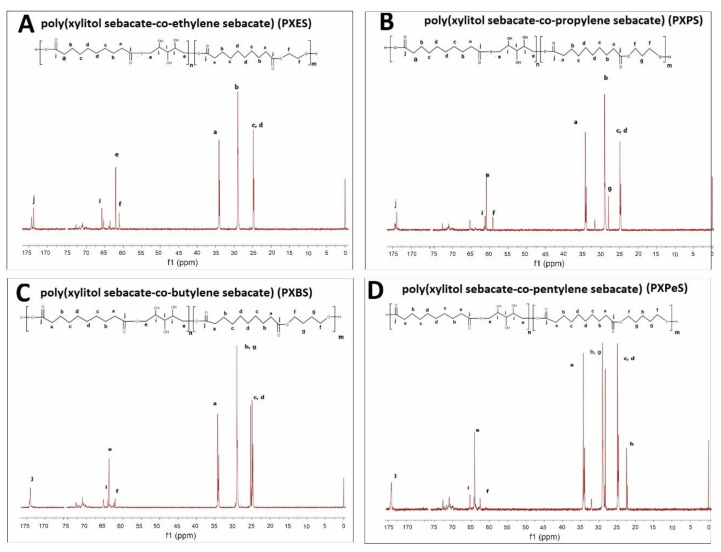
^13^C NMR of following prepolymers: PXES (**A**), PXPS (**B**), PXBS (**C**) PXPeS (**D**) prepolymers.

The prepolymer chemical structure was ascertained by ^1^H NMR and ^13^C NMR. The results are presented in Figure 2, Figure 3, Figure 4, Figure 5.

In ^1^H NMR, the alkyl groups can be linked to the following signals: 2.4–2.6 ppm—CH2 (a), 1.7 ppm—CH2 (g) and CH2 (b), 1.4 ppm—CH2 (h), 1.2 ppm—CH_2_ (c) and CH_2_ (d) Peaks in the range of 3.6 to 4.6 ppm are linked to secondary −OH groups in xylitol. Two signals connected to alkyl groups next to ester bonds can be observed: 4.2 ppm—CH_2_ (f) (diol-acid ester bond), and 4.4 ppm—CH_2_ (e) (xylitol-acid ester bond). By dividing the areas of those two signals, the molar composition was calculated.

In ^13^C NMR, the alkyl groups can be linked to the following signals: 34 ppm—CH_2_ (a), 29 ppm—CH_2_ (g) and CH_2_ (b), 25 ppm—CH_2_ (c) and CH_2_ (d), 22 ppm—CH_2_ (h). The CH_2_OH (i) groups in xylitol result in a signal at 65 ppm. Two signals connected to alkyl groups next to ester bonds can be observed: 64 ppm—CH_2_ (f) (diol-acid ester bond), and 62 ppm—CH_2_ (e) (xylitol-acid ester bond). The carbonyl group results in a signal at 172 ppm.

### 4.2. Fourier Transform Infrared Spectroscopy (FTIR)

**Figure 6:**
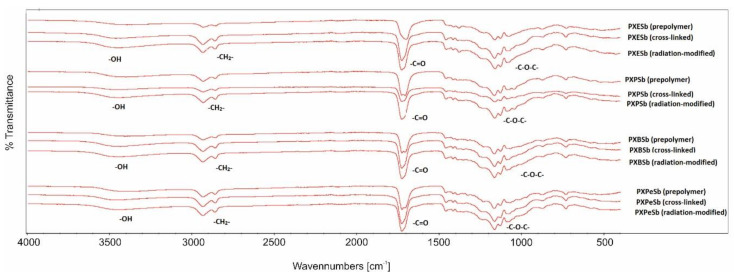
FTIR spectra of PXESb, PXPSb, PXBSb, PXPeSb prepolymers, cross-linked polymers, and radiation-modified polymers.

**Figure 7:**
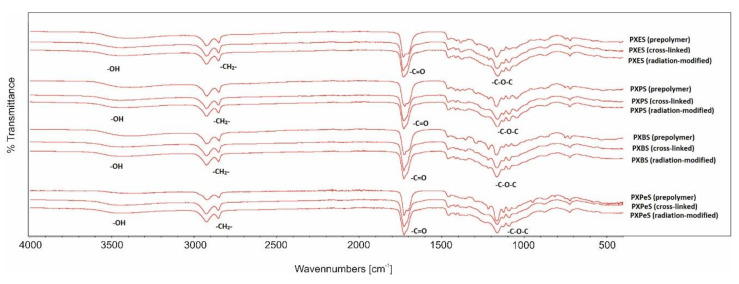
FTIR spectra of PXES, PXPS, PXBS, prepolymers, cross-linked polymers, and radiation-modified polymers.

The FTIR spectra are presented in Figure 6 and Figure 7. The presence of four groups typical for polyesters based on sugar alcohols can be ascertained: –C–O–C groups give a signal at 1170 cm^−1^, C=O groups result in a signal at 1725 cm^−1^, CH_2_ groups generate a signal at 2930 cm^−1^, and free –OH groups produce a signal at 3450 cm^−1^. A lack of polymer-deterioration due to e-beam treatment can be ascertained by the absence of significant differences between the spectra of materials pre and post radiation-modification.

The comparison of prepolymer spectra and cross-linked polymers spectra shows a small increase of –C–O–C signal intensities and decrease of –OH signal intensities. This is due to the formation of ester bonds between –OH groups and unreacted molecules of dicarboxylic acids, which binds the chains together and creates cross-links.

### 4.3. Thermal Properties: Differential Scanning Calorimetry (DSC)

**Figure 8:**
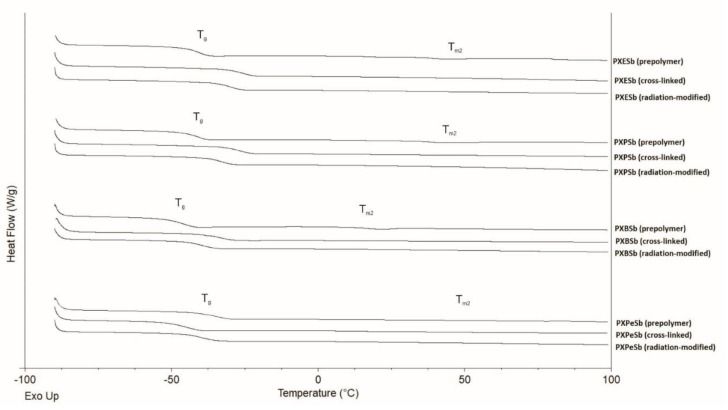
DSC thermograms (first-heating) of PXESb, PXPSb, PXBSb, and PXPeSb prepolymers, cross-linked polymers, and radiation-modified polymers.

**Figure 9:**
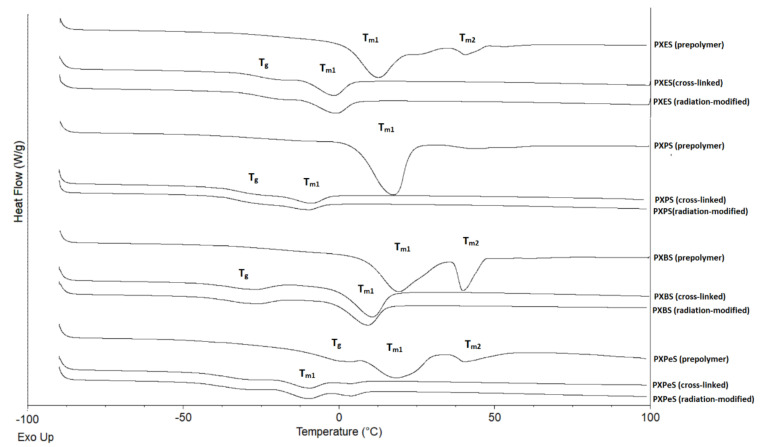
DSC thermograms (first-heating) of PXES, PXPS, PXBS, PXPeS prepolymers, cross-linked polymers, and radiation-modified polymers.

We conducted an analysis and comparison of the thermal properties of elastomers obtained by using different combinations of diols and dicarboxylic acids, and examined how those properties were affected by radiation modification using differential scanning calorimetry. The results are presented in Figure 8 and Figure 9 and in Table 2.

All samples (except for PXES, PXPS, and PXBS prepolymers) exhibit a glass transition temperature.

Melting temperatures are present for all prepolymers. Although the melting temperature is present, the polymers as a whole do not melt. The melting occurs only in small crystalline regions trapped within the amorphous elastomer network. T_m1_ is the result of the melting of poly(xylitol dicarboxylate) segments, and T_m2_ is the result of the melting of poly(diol dicarboxylate) segments. In all elastomers, the melting temperature T_m2_ disappears after the cross-linking process, In case of sebacic-acid-based elastomers, the melting temperature T_m1_ shifts in the direction of lower values as a result of the cross-linking reaction. the melting enthalpy also decreases.

For elastomers based on suberic acid (except for PXPeSb), glass transition increases due to the cross-linking, and then decreases again as a result of e-beam treatment. This is due to the chain mobility decreasing as a result of cross-linking, and then increasing again as a result of radiation modification. For elastomers based on sebacic acid, glass transition is not present in prepolymers except for PXPeSb.

In sebacic-acid-based elastomers, the glass transition temperature stays within a similar range for both cross-linked and e-beam-treated materials, but the heat capacity increases as a result of radiation modification.

The glass transition temperature also decreases for all the materials with the increase in monomer-chain-length due to the chain mobility decreasing.

### 4.4. Dynamic Thermomechanical Analysis (DMTA)

**Figure 10:**
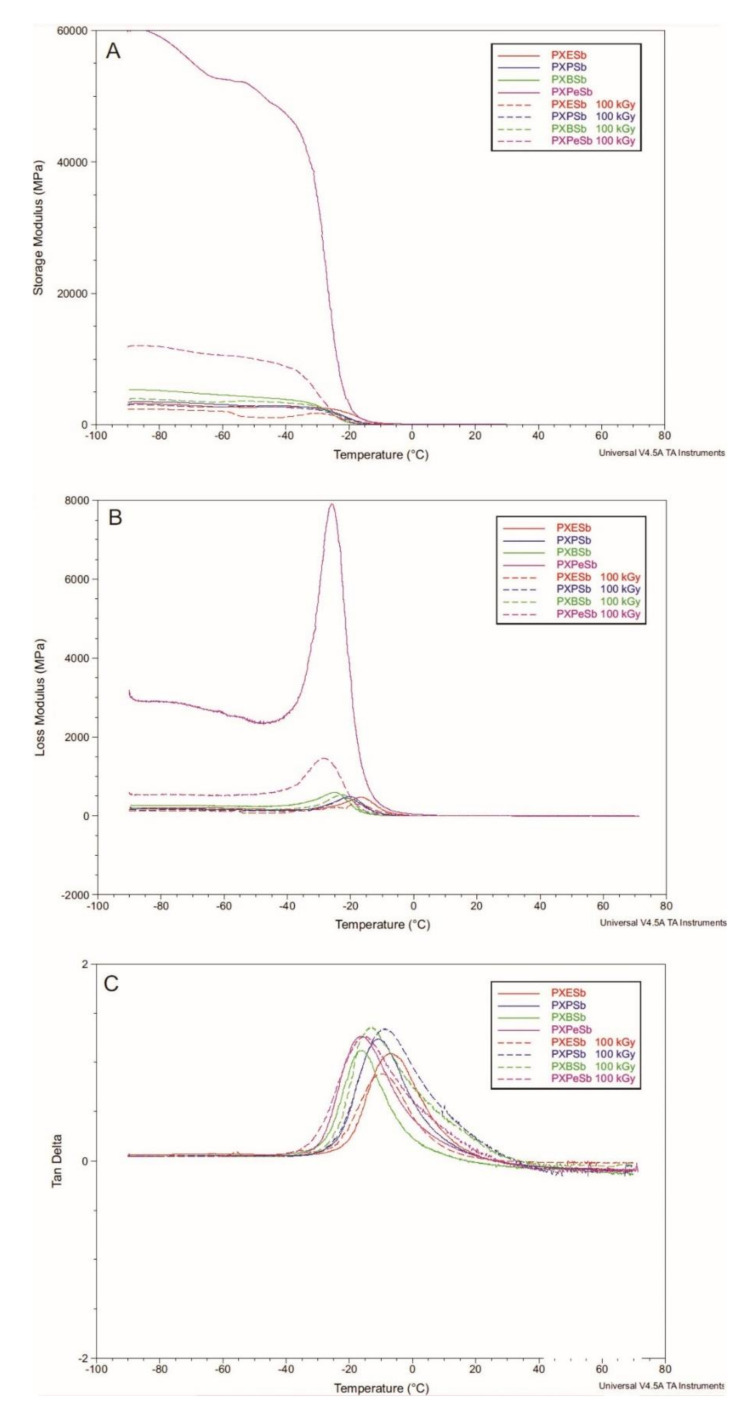
(**A**) E′ (storage modulus), (**B**) E″ (loss modulus), and (**C**) loss tangent tested by DMTA for PXESb, PXPSb, PXBSb, and PXPeSb.

**Figure 11:**
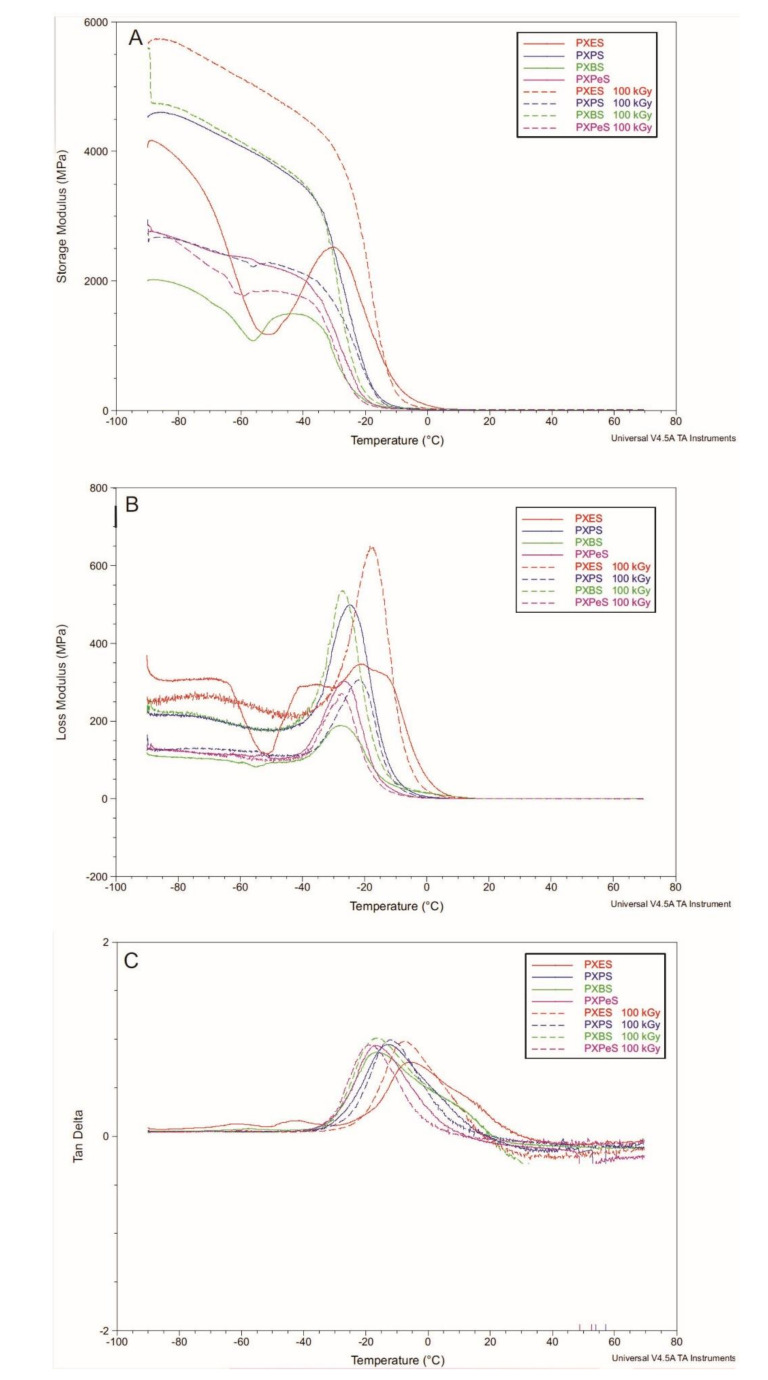
(**A**) E′ (storage modulus), (**B**) E″ (loss modulus), and (**C**) loss tangent tested by DMTA for PXES, PXPS, PXBS, and PXPeS.

The relaxation behavior of PXESb, PXPSb, PXBSb, and PXPeSb (Figure 10), and PXES, PXPS, PXBS, and PXPeS (Figure 11), was tested with DMTA. E′ (storage modulus), E″ (loss modulus), and tan delta (loss tangent) were measured as a function of temperature. Materials in a temperature range between −90 and −30 °C are in a glassy state and undergo viscoelastic elastation in a temperature range between −30 and 0 °C. This is associated with glass transition and can be determined by a significant diminishment of storage modulus and presence of loss modulus and loss tangent function maxima. The temperature of glass transition shifts in the direction of lower temperatures with the increase of the diol chain length. Those results coincide with DSC-determined glass transition temperature. PXPeSb material is characterized by substantially higher loss and storage modulus values than other materials, which decrease as a result of radiation modification due to the stabilization of the structure.

PXES and PXBS materials show a significant decrease followed by an increase of storage modulus at −60 °C, which is before glass transition, and in case of PXES, a second peak of the loss function at −60 °C is present. This can be linked to non-cross-linked end-chain-fragments trapped within an amorphous cross-linked phase. The structure stabilizes after e-beam treatment, and this sharp decrease is no longer present. For radiation-modified PXPS and PXPeS materials, however, a slight decrease of storage modulus before glass transition can be seen, which was not present in non-modified materials. This could be an indication of some degradation of those materials taking place.

This change in storage modulus after e-beam treatment is mirrored by the change in stress at break determined by tensile tests. PXES and PXBS materials, which stabilize as a result of radiation, are also characterized by an increase of stress at break as a result of e-beam treatment. However, for PXPS and PXPeS materials, which show signs of degradation as a result of irradiation, stress at break decreases.

### 4.5. Mechanical Properties

**Figure 12:**
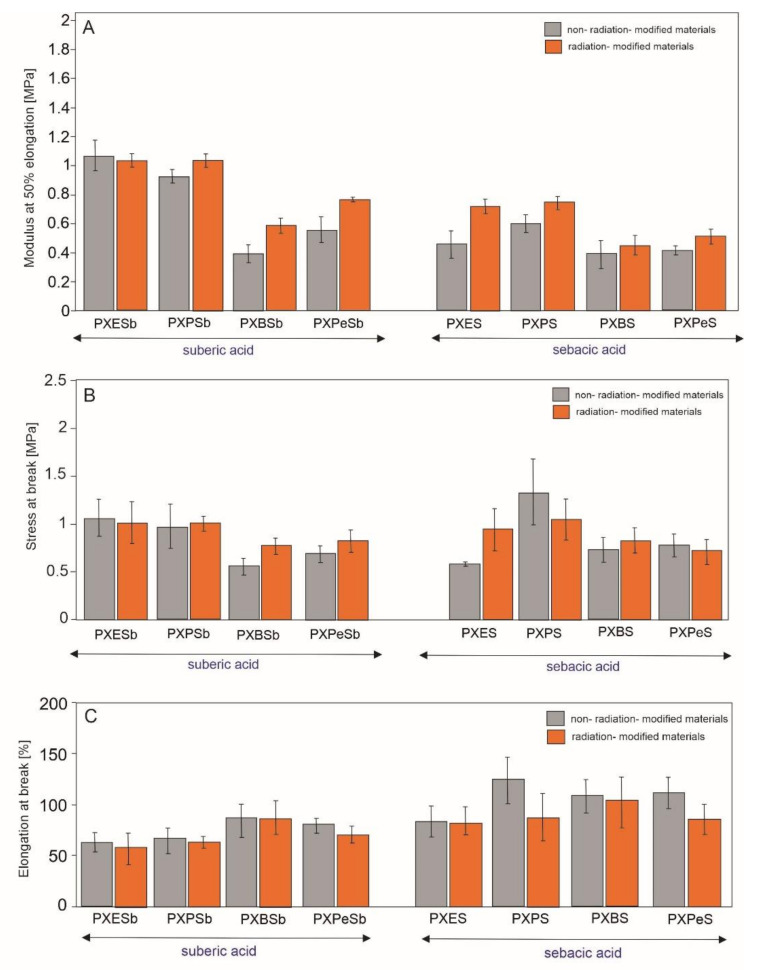
Mechanical properties of PXESb, PXPSb, PXBSb, PXPeSu and PXES, PXPS, PXBS, PXPeS non-radiation-modified materials (gray) and radiation-modified materials (orange): tangent modulus at 50% elongation (**A**), stress at break (**B**), and elongation at break (**C**).

Tensile tests were performed to test and compare mechanical properties of xylitol-based elastomers synthesized with combination of different diols and dicarboxylic acids Another goal was determining whether performing the radiation modification results in a beneficial alteration of elastomer properties. The results of tensile tests are presented in Figure 12 and Table 1. PXPS has the highest elongation at stress at break, while PXESb has the highest modulus at 50% elongation. Radiation modification leads to an increase in modulus at 50% elongation for all the materials with the exception of PXESb, and the increase of stress at break for PXPSb, PXBSb, PXPeSb, PXES, and PXBS. Elongation at break decreases due to e-beam treatment, which is typical for radiation modification [1]. Overall, all materials except for PXESb exhibit some improvement in their properties as a result of e-beam treatment.

### 4.6. Gel Fraction

**Figure 13:**
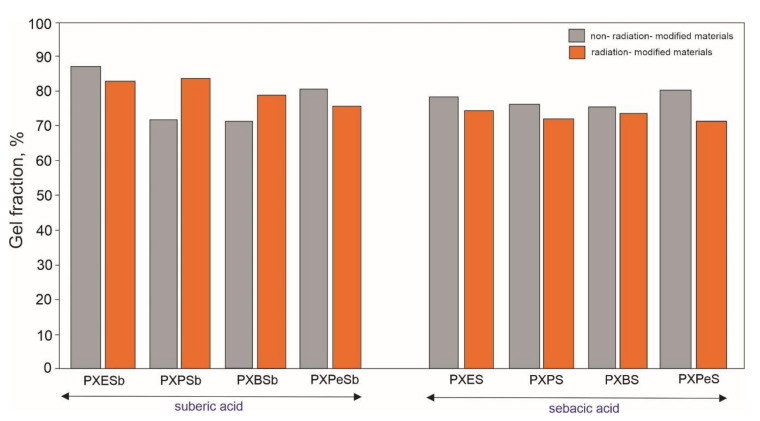
Gel fraction results for PXESb, PXPSb, PXBSb, PXPeSb and PXES, PXPS, PXBS, PXPeS non-radiation-modified materials (gray) and radiation-modified materials (orange).

The results of gel fraction determination are presented in Figure 13. The gel fraction content of non-radiation-modified materials is within a range of 70–88%, with PXESb having the highest gel fraction content. The gel fraction content increases only for PXPSb and PXBSb as a result of radiation modification, while it decreases for the rest of the materials. There is no correlation between the change of gel content and other properties of the elastomers.

### 4.7. Biodegradation

**Figure 14:**
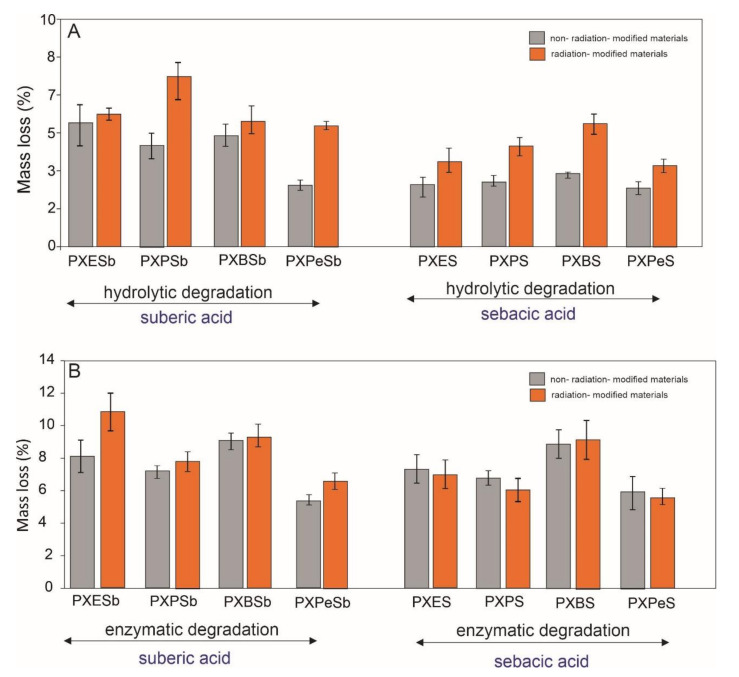
Hydrolytic (**A**) and enzymatic degradation (**B**) of PXESb, PXPSb, PXBSb, PXPeSb and PXES, PXPS, PXBS, PXPeS non-radiation-modified materials (gray) and radiation-modified materials (orange).

Biodegradation tests were performed to test how the monomer chain length and subsequent e-beam treatment affect the materials’ susceptibility to both enzymatic and hydrolytic degradation. The results are presented in Figure 14. Materials based on suberic acid and ethanediol-1,3-propanediol-, and 1,4-butanediol are most susceptible to hydrolytic degradation, with mass loss in the range of 4–5.5%. Mass loss of the rest of the materials is significantly lower and about 3%. Addition of the enzyme to the degradation solution also leads to significant increase in mass loss. Materials based on 1,4-butanediol are most susceptible to such degradation, while materials based on 1,5-pentanediol are least susceptible. There is a significant increase in the degradation susceptibility of materials due to e-beam treatment.

### 4.8. Thermogravimetric Analysis (TGA)

**Figure 15:**
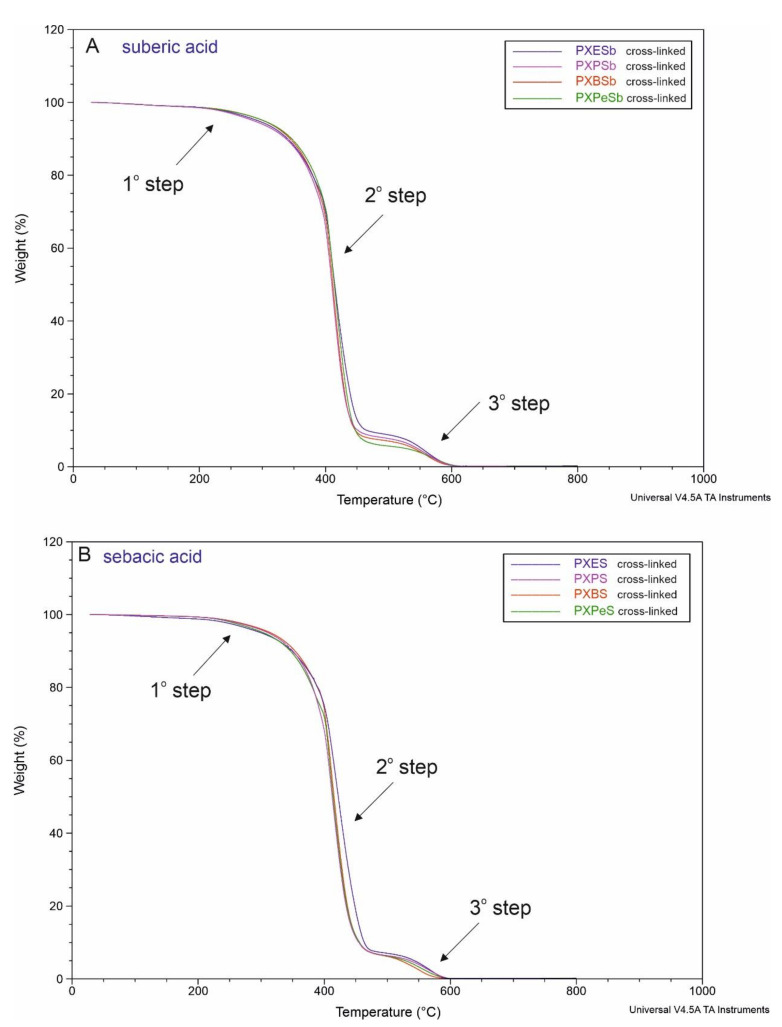
Thermogravimetric analysis (TGA) of PXESb, PXPSb, PXBSb, PXPeSb (**A**) and PXES, PXPS, PXBS, PXPeS (**B**) after synthesis.

The thermal stability of the elastomers was tested by TGA. The results are presented in Figure 15. All materials have thermal stability up to 250 °C, which is both significantly higher than the foreseeable temperature of use and the temperature during synthesis and cross-linking. Materials start to degrade above 250 °C. Three steps of material degradation can be observed. Step 1 at about 250–300 °C leads to about 3.2% mass loss and is possibly due to the impurities present in the materials. Step 2 starting at 300 °C and ending at 470 °C is due to the decomposition of the polymer, and leads to 86% mass loss. Charred remains of the polymer remain relatively stable between 470 and 570 °C, and evaporate completely at temperatures between 570 and 600 °C (step 3) The function of mass loss versus temperature is similar for all the materials.

## 5. Conclusions

Eight elastomers based on two dicarboxylic acids, namely suberic and sebacic acid, and four different diols were synthesized. Materials were modified by e-beam treatment, and a positive effect of such modification on mechanical characteristics was confirmed for all the materials except PXESb. ^1^H NMR and ^13^C NMR delivered information about the polymer structure and allowed the calculation of a molar composition of poly(xylitol dicarboxylate) to poly(diol dicarboxylate) segments of obtained materials. FTIR was used to confirm the cross-linking process taking place and to determine that the polymer structure does not deteriorate as a result of radiation modification. Thermal properties were tested by DSC and DMTA analyses, and the results obtained from these two methods complement each other well. Elastomers were also determined to have good thermal stability by TGA analysis. Materials were also characterized by good susceptibility to both enzymatic and hydrolytic degradation, which was further enhanced by e-beam treatment. Overall, it was determined that the properties of poly(xylitol-dicarboxylate-co-diol dicarboxylate) can be tailored for a specific application. This application could foreseeably be polymeric boluses for radiotherapy treatment. Materials described in this work are good candidates for such application because of their very good response to the influence of radiation, wide range of possible to obtain mechanical properties, thermal stability, and ease of disposal (due to biodegradability).

## Figures and Tables

**Table 1:** Properties and molar composition of poly(xylitol dicarboxylate-co-diol dicarboxylate) pre and post e-beam treatment.

Material	Molar Composition (Ratio of poly(xylitol dicarboxylate) Blocks to poly(diol dicarboxylate) Blocks) Determined by ^1^H NMR for Prepolymers	Stress at Break (MPa)	Elongation at Break (%)	Modulus at 50% Elongation (MPa)
PXESb	1.33	1.01 +/− 0.23	62.77 +/− 9.34	1.08 +/− 0.19
PXESb (radiation-modified)	-	1.06 +/− 0.22	59.1 +/− 14.9	1.03 +/− 0.05
PXPSb	0.51	0.96 +/− 0.23	67.00 +/− 12.54	0.92 +/− 0.05
PXPSb (radiation-modified)	-	1 +/− 0.078	63.62 +/− 3.33	1.04 +/− 0.05
PXBSb	0.27	0.56 +/− 0.08	88.14 +/− 15.77	0.40 +/− 0.07
PXBSb (radiation-modified)	-	0.78 +/− 0.08	88 +/− 16.4	0.58 +/− 0.05
PXPeSb	0.47	0.70 +/− 0.09	81.68 +/− 7.24	0.56 +/− 0.09
PXPeSb (radiation-modified)	-	0.83 +/− 0.12	72 +/− 8.4	0.764 +/− 0.02
PXES	1.02	0.58 +/− 0.02	84.40 +/− 15.43	0.45 +/− 0.09
PXES (radiation-modified)	-	0.94 +/− 0.22	82.3 +/− 13.9	0.72 +/− 0.05
PXPS	0.5	1.33 +/− 0.34	125.53 +/− 23.19	0.60 +/− 0.06
PXPS (radiation-modified)	-	1.06 +/− 0.22	89 +/− 23.09	0.74 +/− 0.05
PXBS	0.44	0.72 +/− 0.13	107.87 +/− 16.06	0.39 +/− 0.10
PXBS (radiation-modified)	-	0.82 +/− 0.13	105 +/− 24.9	0.45 +/− 0.07
PXPeS	0.45	0.78 +/− 0.13	112.9 +/− 15.28	0.42 +/− 0.03
PXPeS (radiation-modified)	-	0.68 +/− 0.07	90.65 +/− 19.35	0.54 +/− 0.11

where E_100%: Modulus at 100% elongation, E_50%: Modulus at 50% elongation, ε: Elongation at break, σ_r_: Stress at break.

**Table 2:** DSC thermal data of poly(xylitol dicarboxylate-co-diol dicarboxylate) before and after irradiation.

Material	Glass Transition Temperature T_g_ (°C)	Change in Heat Capacity ∆C_p_ (J/g °C)	Melting Temperature T_m1_ (°C)	Melting Enthalpy H_m1_ (J/g)	Melting Temperature T_m2_ (°C)	Melting Enthalpy H_m2_ (J/g)
PXESb prepolymer	−40.3	0.69	-	-	45.02	1.40
PXESb cross-linked	−25.6	0.56	-	-	-	-
PXESb (radiation-modified)	−29.3	0.57	-	-		
PXPSb prepolymer	−40.6	0.61	-	-	44.70	2.20
PXPSb cross-linked	−26.3	0.51	-	-	-	-
PXPSb (radiation-modified)	−32.9	0.53	-	-	-	-
PXBSb prepolymer	−45.9	0.67	-	-	21.6	1.31
PXBSb cross-linked	−32.9	0.47	-	-	-	-
PXBSb (radiation-modified)	−39.6	0.55				
PXPeSb prepolymer	−34.5	0.51	-	-	53.9	0.42
PXPeSb cross-linked	−44.9	0.68	-	-	-	-
PXPeSb (radiation-modified)	−40	0.54				
PXES prepolymer	-	-	12.4	33.9	40.5	3.46
PXES cross-linked	−26	0.49	−1.8	10.3	-	-
PXES (radiation-modified)	−25.3	0.51	−1.09	9.8		
PXPS prepolymer	-	-	17.1	45.3	-	-
PXPS cross-linked	−32.5	0.40	−8.4	5.1	-	-
PXPS (radiation-modified)	−33.4	0.47	−9.5	3.5		
PXBS prepolymer	-	-	18.9	33.5	39.9	12.6
PXBS cross-linked	−35.5	0.43	10.6	24.6	-	-
PXBS (radiation-modified)	−34.8	0.48	9.16	17.6		
PXPeS prepolymer	−4.3	0.78	19.2	20.1	40.9	5
PXPeS cross-linked	−35.5	0.47	−9.6	7.5	-	-
PXPeS (radiation-modified)	−36.2	0.50	−10.5	4.2	4.3	1.4

where ∆C_p_: Heat capacity change, T_m1_: Melting temperature, T_g_: Glass transition temperature, ∆H_m1_: Melting enthalpy.

## Data Availability

The data presented in this study is available on request from the corresponding authors.
